# Development of a relational workplace social capital scale for Japanese nurses

**DOI:** 10.1186/s12199-020-00879-0

**Published:** 2020-08-12

**Authors:** Kensuke Norikoshi, Toshio Kobayashi, Keiji Tabuchi, Sanae Oriyama

**Affiliations:** 1 Faculty of Nursing, Hiroshima International University, 5-1-1, Hirokoshingai, Kure, Hiroshima, 737-0112 Japan; 2 Doctoral Program in Biomedical and Health Sciences, Hiroshima University, 1-2-3, Kasumi, Minami-ku, Hiroshima, 734-8551 Japan; 3 Department of General Internal Medicine, Ishii Memorial Hospital, 3-102-1, Tada, Iwakuni, Yamaguchi, 741-8585 Japan; 4 Research and Education Faculty, Medical Sciences Cluster, Nursing Science Unit, Kochi University, Okocyokohasu, Nankoku, Kochi 783-8505 Japan; 5 Graduate School of Biomedical and Health Sciences, Hiroshima University, 1-2-3, Kasumi, Minami-ku, Hiroshima, 734-8551 Japan

**Keywords:** Social capital, Nurses, Workplace, Reproducibility of results, Psychometrics

## Abstract

**Background:**

Although nurses’ workplace social capital for a healthy work environment has received considerable attention, few scales about nurses’ workplace social capital are based on the attributes of clinical settings in Japan. This study aims to develop a Relational Workplace Social Capital Scale for Japanese Nurses (RWSCS-JN), which includes bonding, linking, and bridging social capital and assessing its reliability and validity.

**Methods:**

We assessed its reliability and validity using questionnaire survey data collected from 309 nurses in the first survey and 105 nurses in the second survey in four hospitals in Japan. First, we determined the number of factors and items for the RWSCS-JN through the parallel and factor analyses after conducting the item analysis. Then, we confirmed the omega coefficients and intraclass correlation coefficients (ICC) of the RWSCS-JN. Finally, we examined the Pearson product-moment correlation coefficient between the RWSCS-JN score and other variables, including an existing measurement of workplace social capital, work engagement, and turnover intention.

**Results:**

The newly developed RWSCS-JN contained 15 items, comprising three factors as follows: bonding social capital, linking social capital, and bridging social capital. The omega coefficient and the ICC of the RWSCS-JN were 0.90 and 0.85, respectively. The Pearson product-moment correlation coefficient between the RWSCS-JN and the existing scale of the workplace social capital was 0.88 (*p* < 0.01). Furthermore, the Pearson product-moment correlation coefficient between the RWSCS-JN and work engagement was 0.36 (*p* < 0.01) and that of the RWSCS-JN and turnover intention was − 0.40 (*p* < 0.01).

**Conclusions:**

This study suggests that the RWSCS-JN could be sufficiently useful for a healthy work environment in a clinical setting.

## Background

Workplace social capital (WSC) indicates features of social structures such as levels of interpersonal trust and norms of reciprocity and mutual aid, which act as resources for individuals and facilitate collective action. Thus, WSC is equivalent to the definition of social capital [1]. This concept has gained substantial attention in healthcare settings since around 2010s because facilitating nurses’ WSC exerts a positive impact, such as work engagement [2–4], turnover intention [5], emotional exhaustion [6–8], and risk management [9–11].

Nurses’ WSC is defined as nurses’ shared assets and manners of being and knowing that are evident in, and available through, nurses’ networks of social relationships at work [12]. The attributes of nurses’ WSC include a social structure that enables nurses to entirely use their abilities both vertically and horizontally, supported by a sense of security [13].

The components of social capital are diverse based on the following previous research. According to Islam et al. [14], the social capital can be divided into cognitive social capital and structural social capital. Cognitive social capital includes norms, values, attitudes, and beliefs, and structural social capital refers to externally observable aspects of social organization, such as the density of social networks. In addition, the social capital of relational dimension can also be distinguished into horizontal social capital and linking social capital (a network connecting individuals and organizations of different social classes). Conversely, horizontal social capital can be further distinguished as bonding social capital (a homogeneous connection, such as a group member) and bridging social capital (a network connecting heterogeneous people and organizations) [15].

The WSC scale developed by Kouvonen et al. [16] comprises 8 items and 2 factors, structural and cognitive dimension. This scale can also comprise 3 factors: bonding, bridging, and linking social capital depending on the combination of the items and was used in many previous studies targeting various workplaces [3, 17–20]. The Social Capital in Organizations Scale developed by Ernstmann et al. [9] comprises 6 items and 2 factors, perceived common values and trust in the organization; these factors are positioned as cognitive social capital and also used in several previous studies of WSC targeting clinical settings [7, 21, 22]. In addition, the Social Capital of Nursing Scale developed by Sheingold et al. [23] comprises 29 items and 5 factors as follows: external trust, solidarity and empowerment, participation and affiliation, internal trust, solidarity, and harmony; social cohesion with coworkers; and conflict. Moreover, this scale can focus not only on the bonding social capital, such as relationships with nurses in the same unit, but also the bridging social capital and linking social capital; it was also used in a previous study of WSC for nurses [24]. Although these measurements can be elucidated structurally, it is imperative to comprehensively assess whether nurses’ WSC adequately expresses the attributes of Japanese nurses.

In Japan, the Social Capital and Ethical Climate at the Workplace of a Hospital Scale developed by Tei-Tominaga et al. [25, 26] comprises 20 items and 3 factors, social capital in the workplace, exclusive workplace climate, and ethical leadership. In addition, the Bonding Social Capital Scale developed by Eguchi et al. [27] has 6 items and 1 factor. However, elucidating the social structure of nurses’ WSC using these measurements is challenging. As some hospitals have introduced a partnership nursing system in which two nurses care for several patients [28], the robust cooperation among nurses is increasingly essential. Moreover, the significance of cooperation among professions with different specialized backgrounds is largely highlighted, and professionals with different expertise understand each other’s expertise, share patients’ goals, and strive to provide high-quality services. The growing importance of the multidisciplinary approach mandates elucidating the relational workplace social capital such as the bonding social capital, the linking social capital, and the bridging social capital, based on the attributes of nurses’ WSC in Japan.

Hence, this study aims to assess the reliability and validity of a newly developed Relational Workplace Social Capital Scale for Japanese Nurses (RWSCS-JN) including the attributes of nurses’ WSC in Japan. By using this scale to grasp the nurses’ WSC from the relational aspects, it is possible to clarify the effective human resources method for clinical nurses toward the healthy work environment.

## Methods

### Scale development procedure

The procedures to develop the item pool of RWSCS-JN comprised three steps. First, the initial 16 items were gathered from literature review and interview data with 32 nurses [13]. Next, the content validity of the RWSCS-JN was approved by a discussion until consensus was reached with six researchers who have doctoral degrees of medical science or nursing and have research experience for the WSC. Additionally, the face validity of the RWSCS-JN about the arrangement of duplicate items and the appropriateness of expressions were assessed among 10 nurses using a questionnaire. Finally, we discussed the content of the RWSCS-JN again with the researchers and developed the RWSCS-JN comprising 16 items.

### Participants and data collection procedure

We conducted self-administered questionnaire surveys by mail two times using the same procedures at an interval of 2 weeks to evaluate the test-retest reliability of the RWSCS-JN from November 2017 to December 2017. We requested creating an 8-digit ID number, such as the participants’ dates of birth, during the first survey to connect the data collected at different time points while securing the participants’ anonymity. Each questionnaire was distributed to a convenience sampling of 693 nurses in four hospitals by the director of nursing. Of these, 329 nurses responded to the first survey and 127 nurses responded to the second survey; however, we excluded data from the analysis when the answering box was left blank, more than one option was selected when it was not allowed, or if the answer was repeated with the same number. Consequently, valid responses were obtained from 309 nurses (valid response rate, 44.6%) in the first survey and 105 nurses (valid response rate, 15.2%) in the second survey. Inclusion criteria for the participants were as follows: (a) nurses with a minimum experience of 6 months; (b) nurses with manager/chief or staff position; and (c) nurses with full-time or part-time status. Exclusion criteria for the participants was nurses with director/assistant director of nursing position.

### Measures

#### Demographic variables

Based on the previous study of nurses’ WSC [25], we measured the participants’ sex, age, marital status, employment status, years of nursing experience, position, number of hospital beds, and work unit.

#### Nurses’ WSC

We measured the newly developed RWSCS-JN to assess its reliability and validity. The scale comprises 16 items that are scored on a 5-point Likert scale ranging from 1 (never) to 5 (always), with the higher total score indicating higher levels of nurses’ WSC.

When evaluating the test-retest reliability, it is imperative that the state of the construct for the measurement be steady between the two surveys. In addition, it is necessary to measure another measurement, called anchor, which is easy to interpret intuitively besides the measurement evaluated for the reliability [29]. Thus, the anchor was measured as nurses’ WSC in the second survey as follows: “how is the cooperative relationship with others in the workplace compared with the previous survey?” The anchor was scored on a 7-point Likert scale with scores ranging from 1 (very much worse) to 7 (very much improved). Notably, only participants with a score of 3 (no change) were analyzed in the second survey.

#### WSC

We measured the WSC with the Japanese version of the WSC scale [30] to assess the convergent validity. The scale comprises 8 items (e.g., we have a “we are together” attitude; do members of the work unit build on each other’s ideas in order to achieve the best possible outcome?; our supervisor treats us with kindness and consideration). All items are scored on a 5-point Likert scale ranging from 1 (never) to 5 (always), with the higher total score suggesting higher levels of the WSC. These items indicated whether people felt that they are respected, valued, and treated as equals at work, rather than feeling that it was all a matter of seniority in their hierarchy. In this study, the omega coefficient for this scale was 0.89.

#### Work engagement

We measured the work engagement with the Japanese Short Version of the Utrecht Work Engagement Scale [31] to assess the convergent validity because higher nurses’ WSC were associated with higher work engagement [2, 3, 21]. This scale comprises 9 items and 3 factors as follows: vigor, dedication, and absorption (e.g., at my work, I feel bursting with energy; I am enthusiastic about my job; I feel happy when I am working intensely). All items are scored on a 7-point Likert scale ranging from 0 (“never”) to 6 (“always”), with the higher total score indicating high work engagement. In this study, omega coefficient for this scale was 0.93.

#### Turnover intention

We measured the turnover intention with the Turnover Intention Scale [32] to assess the criterion validity because lower turnover intention were associated with higher WSC [5]. This scale comprises 4 items as follows: “As soon as I get another acceptable job I will quit,” “I want to leave this organization very much,” “I think about quitting all the time,” and “I intend to quit this organization someday soon.” All items are scored on a 5-point Likert scale ranging from 1 (“never”) to 5 (“always”), with a higher score of these items suggesting stronger turnover intention. In this study, the omega coefficient for this scale was 0.92.

### Statistical analysis

All statistical analyses were performed using R version 3.4.3 [33] and IBM SPSS Statistics for Windows version 25.0 (SPSS Japan Inc., Tokyo, Japan) with the level of significance set at 5%.

First, after computing descriptive statistics, we considered exclusion items of the RWSCS-JN by calculating the ceiling and floor effect and analyzing the item-total correlation coefficient and the inter-item correlation coefficient. The exclusion criteria of the item were “ceiling and floor effect mean − SD < 1 or mean + SD > 5,” “inter-item correlation coefficient ≥ 0.85” and “item-total correlation coefficient < 0.30.”

Second, we conducted the exploratory factor analysis using the maximum likelihood method with Promax rotation after determining the number of factors by the parallel analysis and minimum average partial method. The items of the RWSCS-JN with factor loadings < 0.4 or an item loaded on one and more factors were dropped from the scales. The sample size required for conducting the factor analysis is 4:1 to 10:1 ratio between the number of participants and the number of items, with a minimum of 100 participants [34]. Thus, the sample size of the present study was adequate.

Third, omega coefficients of the RWSCS-JN were confirmed to assess the internal consistency for reliability. The omega coefficient is one of the best alternatives for estimating internal consistency reliability, as it corrects the underestimation bias of Cronbach’s alpha [35, 36]. In addition, to evaluate the test-retest reliability, the intraclass correlation coefficient (ICC) of the RWSCS-JN was confirmed as a ratio of variances, with ICC ≥ 0.61 considered to indicate substantial reliability and ICC ≥ 0.81 considered to indicate almost perfect reliability [37]. The standard error of measurement (SEM) was estimated by computing the square root of the subject variance of the nurses (SEM_agreement_ = √σ_between measurement_ + σ_residual_) [38]. The larger the SEM, the lower the test reliability and the lesser the precision in the measures and scores obtained [39].

Finally, to assess the criterion and convergent validity, we confirmed the Pearson product-moment correlation coefficient with the WSC, work engagement, and turnover intention, which predicted to be theoretically valid with the RWSCS-JN.

In this study, the normality of each measurement could not be confirmed. However, we conducted Pearson’s correlation analysis and factor analysis since Pearson’s correlation analysis is robust against distribution distortion and normality when the sample size is large (sample size ≥ approximately 65) [40] and factor analysis using the maximum likelihood method has asymptotic normality [41].

### Ethical approval

This study protocol was approved by the Research Ethics Board of the Hiroshima International University (Hiroshima, Japan; Protocol No. 17-016). All participants provided written informed consent about the protection of anonymity and confidentiality for the surveys.

## Results

### Sample characteristics

Participants included 23 males, 286 females, with an average age of 38.2 ± 10.0, an average years of professional experience of 14.7 ± 9.6. Participants including married of 178 nurses, 281 (90.9%) worked as full time, and 247 (79.9%) worked as staff nurse. Thirty-eight participants (12.3%) work at a hospital where have beds of 100 and less, 105 participants (34.0%) work at a hospital where have beds of 100–299. and 166 participants (53.7%) work at a hospital where have beds over 300. More than half of participants (63.8%) worked at the department of internal medicine/surgery (Table 1).

**Table 1:** The demographics of the participants in the first survey

	All participants (*N* = 309)
Sex	
Male	23 (7.4%)
Female	286 (92.6%)
Age (means ± SD)	38.2 ± 10.0
Marital status	
Married	178 (57.6%)
Single	129 (41.7%)
Unknown	2 (0.7%)
Employment status	
Full−time	281 (90.9%)
Part−time	28 (9.1%)
Years of nursing experience (means ± SD)	14.7 ± 9.6
Position	
Staff	247 (79.9%)
Chief	47 (15.2%)
Manager	15 (4.9%)
Number of hospital beds	
<100	38 (12.3%)
100–299	105 (34.0%)
≥ 300	166 (53.7%)
Work unit	
Internal medicine/Surgery	197 (63.8%)
Outpatient	44 (14.2%)
ICU/CCU/Emergency room	37 (12.0%)
Recovery rehabilitation	31 (10.0%)

*ICU* intensive care unit, *CCU* coronary care unit

### Descriptive statistics and item analysis for items of the RWSCS-JN

We observed no ceiling and floor effect. In addition, inter-item correlation coefficient for each item less than 0.85 and item-total correlation coefficient for each item ranged between 0.76 and 0.90; therefore, we observed no items which satisfied the exclusion criteria in the item analysis (Table 2).

**Table 2:** Descriptive statistics and item analysis for items on the RWSCS-JN

No.	Items	M	SD	M + SD	M − SD	Skewness	Kurtosis	SWT^a^	IIC	ITC
1	Nurses in my work unit treats us with thoughtful.	4.08	0.73	4.80	3.35	− 0.63	0.85	0.81	0.18–0.63	0.81
2	We feel protected somehow at our workplace.	3.29	1.02	4.31	2.27	− 0.21	− 0.26	0.90	0.28–0.57	0.90
3	We can receive our supervisor’s support.	3.70	0.89	4.59	2.81	− 0.60	0.17	0.86	0.21–0.72	0.86
4	We can receive other professions' support.	3.53	0.86	4.39	2.67	− 0.36	0.01	0.88	0.27–0.62	0.88
5	We trust my supervisors.	3.68	1.03	4.71	2.65	− 0.69	0.08	0.87	0.13–0.72	0.87
6	We trust other nurses in our work unit.	3.99	0.76	4.74	3.23	− 0.52	0.42	0.83	0.22–0.63	0.83
7	We trust other professions.	3.65	0.79	4.44	2.86	− 0.22	− 0.12	0.86	0.30–0.62	0.86
8	Our supervisors allocate tasks us with fairness.	3.19	0.92	4.11	2.27	− 0.21	− 0.04	0.89	0.22–0.59	0.89
9	In my workplace, we perform tasks by helping each other.	3.87	0.73	4.60	3.15	− 0.68	1.32	0.81	0.29–0.57	0.81
10	We share information about work with other nurses in the same work unit.	3.97	0.62	4.59	3.35	− 0.55	1.91	0.76	0.35–0.57	0.76
11	We share perspectives on nursing with other nurses in the same work unit.	3.41	0.81	4.22	2.61	− 0.44	0.43	0.86	0.19–0.49	0.86
12	We can convey opinions to other nurses on even ground.	3.40	0.81	4.21	2.59	− 0.56	0.54	0.85	0.13–0.49	0.85
13	We share information about work with other professions.	3.68	0.77	4.45	2.91	− 0.68	0.88	0.82	0.22–0.57	0.82
14	We respect the specialities of other professions.	3.97	0.71	4.67	3.26	− 0.40	0.21	0.82	0.19–0.54	0.82
15	We can convey opinions to other professions on even ground.	3.52	0.86	4.39	2.66	− 0.66	0.29	0.85	0.18–0.57	0.85
16	We appreciate each other at our workplace.	3.77	0.77	4.54	3.00	− 0.37	0.40	0.85	0.27–0.47	0.85

*RWSCS-JN* relational workplace social capital scale for Japanese nurses, *M* mean, *SD* standard deviation, *SWT* Shapiro-Wilk test, *IIC* inter-item correlation, *ITC* item-total correlation

^a^The values shown are statistic

### Construct validity

We conducted the parallel analysis to evaluate the number of factors for the 16 items of the RWSCS-JN and the possibility of interpretation in case the 3-factor structure was judged high. Additionally, the components of social capital are diverse [15], and it was possible that the RWSCS-JN had two types of factor structures. One was the aspect of relationship that included bonding, linking, and bridging social capital, and the other was the aspect of resource, such as trust, reciprocity, and information sharing. Consequently, the explanatory factor analysis was conducted assuming the 3-factor structure using the maximum likelihood method with Promax rotation. After repeating the analysis, we excluded item 7 that exhibits with high factor load for multiple factors (Table 3). The ratio to account for the total variance of 15 items with 3 factors before rotation was 62.00%.

**Table 3:** Final factor pattern of the RWSCS-JN

No	Items	Standardized estimates		
		1	2	3
**Bonding social capital (omega coefficient = 0.86)**				
6	We trust other nurses in our work unit.	0.86	0.02	−0.13
1	Nurses in my work unit treats us with thoughtful.	0.83	0.00	−0.18
9	In my workplace, we perform tasks by helping each other.	0.68	0.11	−0.01
16	We appreciate each other at our workplace.	0.56	0.04	0.10
10	We share information about work with other nurses in the same work unit.	0.54	0.07	0.19
11	We share perspectives on nursing with other nurses in the same work unit.	0.50	−0.05	0.20
12	We can convey opinions to other nurses on even ground.	0.50	−0.18	0.29
**Linking social capital (omega coefficient = 0.86)**				
5	We trust my supervisors.	−0.07	0.92	−0.06
3	We can receive my supervisor's support.	−0.08	0.91	0.00
8	Our supervisors allocate tasks us with fairness.	0.15	0.60	0.03
2	We feel protected somehow at our workplace.	0.18	0.57	0.05
**Bridging social capital (omega coefficient = 0.79)**				
13	We share information about work with other professions.	−0.02	−0.03	0.85
15	We can convey opinions to other professions on even ground.	−0.09	−0.03	0.78
14	We respect the specialities of other professions.	0.07	0.03	0.57
4	We can receive other professions' support.	0.03	0.28	0.49

Note: Omega coefficient of the whole RWSCS-JN was 0.90

*RWSCS-JN* relational workplace social capital scale for Japanese nurses

Factor 1, bonding social capital comprised 7 items was named from the aspect of exchanging resources, such as trust, reciprocity, and information sharing among nurses. Factor 2, linking social capital comprised 4 items was named from the aspect of exchanging resources, such as trust and support, between nurses and supervisors. Factor 3, bridging social capital which comprised 4 items was named from the aspect of exchanging resources, such as information sharing and support, between nurses and other professions.

### Reliability

Table 3 also provides omega coefficients of the RWSCS-JN. Of note, omega coefficients for each factor ranged between 0.79 and 0.86. The overall omega coefficient of the scale was 0.90.

Table 4 provides the ICC and SEM of the RWSCS-JN. The ICC among the factors of the RWSCS-JN ranged between 0.76 and 0.87. The overall ICC of the scale was 0.85 (95% confidence interval: 0.78–0.90, *p* < 0.01). The SEM among the factors of the RWSCS-JN ranged between 1.49 and 2.08. The overall SEM of the scale was 3.83.

**Table 4:** Test-retest reliability of the RWSCS-JN

	Difference score (SD)	ICC (95%CI)	SEM
Whole RWSCS-JN	3.99 (3.65)	0.85 (0.78–0.90)	3.83
Factor of RWSCS-JN			
Bonding social capital	2.09 (2.08)	0.84 (0.77–0.89)	2.08
Linking social capital	1.62 (1.40)	0.87 (0.80–0.91)	1.51
Bridging social capital	1.49 (1.49)	0.76 (0.64–0.84)	1.49

*RWSCS-JN* relational workplace social capital scale for Japanese nurses, *SD* standard deviation, *ICC* intraclass correlation coefficients, *CI* confidence interval, *SEM* standard error of measurement

### Criterion and convergent validity

Table 5 provides the correlation coefficient between the RWSCS-JN score and other scales. The Pearson product-moment correlation coefficient of the RWSCS-JN and WSC was 0.88. In addition, the Pearson product-moment correlation coefficient of the RWSCS-JN and work engagement was 0.36, RWSCS-JN and turnover intention was − 0.40.

**Table 5:** Correlation between the RWSCS-JN score and other scales

	WSC	Work engagement	Turnover intention
Whole RWSCS-JN	0.88**	0.36**	−0.40**
Factor of RWSCS-JN			
Bonding social capital	0.81**	0.27**	−0.29**
Linking social capital	0.76**	0.35**	−0.44**
Bridging social capital	0.58**	0.28**	−0.27**

*RWSCS-JN* relational workplace social capital scale for Japanese nurses, *WSC* workplace social capital

**p< 0.01, Pearson product-moment correlation analysis

## Discussion

We assessed the reliability and validity of the newly developed RWSCS-JN, including the attributes of nurses’ WSC in Japan [13]. On checking the consensus-based standards for the selection of health measurement instruments [29], this study established that the reliability coefficient satisfied the criteria and the RWSCS-JN was sufficiently reliable. In addition, it suggested that the RWSCS-JN was sufficiently valid as the correlation between the RWSCS-JN and the others was statistically established, and the adaptation of a 3-factor structure was validated by the exploratory factor analysis.

The omega coefficient and the ICC of the RWSCS-JN were 0.90 and 0.85, respectively. Some studies evaluated good internal consistency for reliability when omega coefficients were greater than 0.7 [35, 42]. Moreover, ICC ≥ 0.81 is considered to indicate almost perfect test-retest reliability [37]. Consequently, the RWSCS-JN was considered to have good internal consistency and test-retest reliability.

The RWSCS-JN comprises 15 items and 3 factors as follows: bonding social capital, linking social capital, and bridging social capital. Although these factors were similar to previous studies [16], in Japan, the significance of multidisciplinary approach is advocated; it is useful to be able to grasp quantitatively not only how to connect among nurses but also how to connect nurses and other professions. Additionally, this study suggested a positive correlation between the RWSCS-JN and work engagement, and a negative correlation was suggested between the RWSCS-JN and the turnover intention. These coincide with previous studies using other WSC scales [2–5] and provide evidence for the validity of the RWSCS-JN.

The turnover rate for full-time nursing staff in the fiscal year 2019 was 10.7% [43], which has been almost flat since the fiscal year 2010, and effects such as the enhancement of various types of employment and promotion of work-life balance are inferred. However, based on the “2017 Survey on labor situation of nursing staff” by the Japan Federation of Medical Worker’s Unions [44], and compared with the previous survey results, it appears that labor shortage became more serious, the acquisition rate for annual paid leave declined, the night shift/shift work facts such as overwork and exhaustion are clear, and the working environment surrounding nurses remains in a difficult situation. These variables may be most relevant from a practical point of view especially for nursing managers who are in charge of human resources management, since the high RWSCS-JN was the correlation with high work engagement and low turnover intention. For example, if nursing managers want to decrease turnover intention in the sampled hospitals, they can apply for a training program to increase the WSC. On the other hand, Oksanen et al. [45] reported the positive correlation between WSC and turnover rate for temporary employees. Therefore, in future studies, it is necessary to further examine the conditions that affect the relationship between WSC and turnover rate/intention.

The findings are difficult to generalize the results of this study because of convenience sampling, such as the sample size was small and applied to a limited region. In the future, it is necessary to increase the accuracy of the developed scale by conducting surveys nationwide and verifying these validity for new groups of nurses.

## Conclusions

This study proposes the RWSCS-JN with 15 items and 3 factors, including bonding social capital, linking social capital, and bridging social capital. In addition, the study establishes the reliability and validity of the scale.

Nursing managers can visually reveal how to relate with others including supervisors and other professions, and it is useful that the RWSCS-JN can be grasp-based on the social structure of clinical settings in Japan. Additionally, this study suggested a negative correlation between the RWSCS-JN and the turnover intention. It was suggested that nursing managers could contribute to decrease turnover intention by applying a training program to increase the WSC.

## Data Availability

The datasets used and analyzed during the current study are available from the corresponding author on reasonable request.

## Abbreviations

RWSCS-JN: Relational workplace social capital scale for Japanese nurses
WSC: Workplace social capital
ICC: Intraclass correlation coefficients
SEM: Standard error of measurement
ICU: Intensive care unit
CCU: Coronary care unit
M: Mean
SD: Standard deviation
SWT: Shapiro-Wilk test
IIC: Inter-item correlation
ITC: Item-total correlation
CI: Confidence interval

## References

[1] Kawachi I, Kennedy B, Lochner K, Prothrow-Stith D. Social capital, income inequality, and mortality. Am J Public Health. 1997. 10.2105/ajph.87.9.1491. 10.2105/ajph.87.9.1491 PMC1380975 9314802

[2] Stromgren M, Eriksson A, Bergman D, Dellve L. Social capital among healthcare professionals: a prospective study of its importance for job satisfaction, work engagement and engagement in clinical improvements. Int J Nurs Stud. 2016. 10.1016/j.ijnurstu.2015.07.012. 10.1016/j.ijnurstu.2015.07.012 26315780

[3] Fujita S, Kawakami N, Ando E, Inoue A, Tsuno K, Kurioka S, et al. The association of workplace social capital with work engagement of employees in health care settings: a multilevel cross-sectional analysis. J Occup Environ Med. 2016. 10.1097/JOM.0000000000000605. 10.1097/JOM.0000000000000605 26949876

[4] Jutengren G, Jaldestad E, Dellve L, Eriksson A. The potential importance of social capital and job crafting for work engagement and job satisfaction among health-care employees. Int J Environ Res Public Health. 2020. 10.3390/ijerph17124272. 10.3390/ijerph17124272 PMC7344872 32549346

[5] Iida M, Watanabe K, Ando E, Tsuno K, Inoue A, Kurioka S, et al. The association between unit-level workplace social capital and intention to leave among employees in health care settings: a cross-sectional multilevel study. J Occup Environ Med. 2020. 10.1097/JOM.0000000000001847. 10.1097/JOM.0000000000001847 32149939

[6] Kowalski C, Driller E, Ernstmann N, Alich S, Karbach U, Ommen O, et al. Associations between emotional exhaustion, social capital, workload, and latitude in decision-making among professionals working with people with disabilities. Res Dev Disabil. 2010. 10.1016/j.ridd.2009.10.021. 10.1016/j.ridd.2009.10.021 20004075

[7] Kowalski C, Ommen O, Driller E, Ernstmann N, Wirtz MA, Köhler T, et al. Burnout in nurses—the relationship between social capital in hospitals and emotional exhaustion. J Clin Nurs. 2010. 10.1111/j.1365-2702.2009.02989.x. 10.1111/j.1365-2702.2009.02989.x 20384668

[8] Ansmann L, Hower KI, Wirtz MA, Kowalski C, Ernstmann N, McKee L, et al. Measuring social capital of healthcare organizations reported by employees for creating positive workplaces—validation of the SOCAPO-E instrument. BMC Health Serv Res. 2020. 10.1186/s12913-020-05105-9. 10.1186/s12913-020-05105-9 PMC7106807 32234055

[9] Ernstmann N, Ommen O, Driller E, Kowalski C, Neumann M, Bartholomeyczik S, et al. Social capital and risk management in nursing. J Nurs Care Qual. 2009. 10.1097/NCQ.0b013e3181b14ba5. 10.1097/NCQ.0b013e3181b14ba5 19755881

[10] Virtanen M, Kurvinen T, Terho K, Oksanen T, Peltonen R, Vahtera J, et al. Work hours, work stress, and collaboration among ward staff in relation to risk of hospital-associated infection among patients. Med Care. 2009. 10.1097/MLR.0b013e3181893c64. 10.1097/MLR.0b013e3181893c64 19194334

[11] Jafari M, Pourtaleb A, Khodayari-Zarnaq R. The impact of social capital on clinical risk management in nursing: a survey in Iranian public educational hospitals. Nurs open. 2018. 10.1002/nop2.141. 10.1002/nop2.141 PMC6056430 30062021

[12] Read EA. Workplace social capital in nursing: an evolutionary concept analysis. J Adv Nurs. 2014. 10.1111/jan.12251. 10.1111/jan.12251 24103033

[13] Norikoshi K, Kobayashi T, Tabuchi K. A qualitative study on the attributes of nurses' workplace social capital in Japan. J Nurs Manag. 2018. 10.1111/jonm.12525. 10.1111/jonm.12525 28944981

[14] Islam MK, Merlo J, Kawachi I, Lindström M, Gerdtham U-G. Social capital and health: does egalitarianism matter? A literature review. Int J Equity Health. 2006. 10.1186/1475-9276-5-3. 10.1186/1475-9276-5-3 PMC1524772 16597324

[15] Acquaah M, Amoako-Gyampah K, Nyathi NQ. Measuring and valuing social capital: a systematic review. Network for Business Sustainability South Africa. 2014; https://static1.squarespace.com/static/5d5156083138fd000193c11a/t/5d6405e7c1dc4300019156a1/1566836207405/NBS-SA-Social-Capital-ER.pdf.

[16] Kouvonen A, Kivimaki M, Vahtera J, Oksanen T, Elovainio M, Cox T, et al. Psychometric evaluation of a short measure of social capital at work. BMC Public Health. 2006. 10.1186/1471-2458-6-251. 10.1186/1471-2458-6-251 PMC1618843 17038200

[17] Firouzbakht M, Tirgar A, Oksanen T, Kawachi I, Hajian-Tilaki K, Nikpour M, et al. Workplace social capital and mental health: a cross-sectional study among Iranian workers. BMC Public Health. 2018. 10.1186/s12889-018-5659-3. 10.1186/s12889-018-5659-3 PMC6019288 29940919

[18] Hori D, Takao S, Kawachi I, Ohtaki Y, Andrea C-S, Takahashi T, et al. Relationship between workplace social capital and suicidal ideation in the past year among employees in Japan: a cross-sectional study. BMC Public Health. 2019. 10.1186/s12889-019-7244-9. 10.1186/s12889-019-7244-9 PMC6617579 31288766

[19] Pekurinen V, Välimäki M, Virtanen M, Kivimäki M, Vahtera J. Work stress and satisfaction with leadership among nurses encountering patient aggression in psychiatric care: a cross-sectional survey study. Adm Policy Ment Health. 2019. 10.1007/s10488-018-00919-6. 10.1007/s10488-018-00919-6 30684111

[20] Kristman VL, Shaw WS, Reguly P, Williams-Whitt K, Soklaridis S, Loisel P. Supervisor and organizational factors associated with supervisor support of job accommodations for low back injured workers. J Occup Rehabil. 2017. 10.1007/s10926-016-9638-1. 10.1007/s10926-016-9638-1 PMC4980120 27032398

[21] Van Bogaert P, Van Heusden D, Timmermans O, Franck E. Nurse work engagement impacts job outcome and nurse-assessed quality of care: model testing with nurse practice environment and nurse work characteristics as predictors. Front Psychol. 2014. 10.3389/fpsyg.2014.01261. 10.3389/fpsyg.2014.01261 PMC4230203 25431563

[22] Van Bogaert P, Kowalski C, Weeks SM, Van Heusden D, Clarke SP. The relationship between nurse practice environment, nurse work characteristics, burnout and job outcome and quality of nursing care: a cross-sectional survey. Int J Nurs Stud. 2013. 10.1016/j.ijnurstu.2013.05.010. 10.1016/j.ijnurstu.2013.05.010 23777786

[23] Sheingold BH, Sheingold SH. Using a social capital framework to enhance measurement of the nursing work environment. J Nurs Manag. 2013. 10.1111/jonm.12127. 10.1111/jonm.12127 23865931

[24] Shin JI, Lee E. The effect of social capital on job satisfaction and quality of care among hospital nurses in South Korea. J Nurs Manag. 2016. 10.1111/jonm.12401. 10.1111/jonm.12401 27197700

[25] Tei-Tominaga M, Nakanishi M. The influence of supportive and ethical work environments on work-related accidents, injuries, and serious psychological distress among hospital nurses. Int J Environ Res Public Health. 2018. 10.3390/ijerph15020240. 10.3390/ijerph15020240 PMC5858309 29385044

[26] Tei-tominaga M, Nakanishi M. Factors related to turnover intentions and work-related injuries and accidents among professional caregivers: a cross-sectional questionnaire study. Environ Health Prev Med. 2020. 10.1186/s12199-020-00863-8. 10.1186/s12199-020-00863-8 PMC7320545 32590934

[27] Eguchi H, Tsutsumi A, Inoue A, Odagiri Y. Psychometric assessment of a scale to measure bonding workplace social capital. PLoS One. 2017. 10.1371/journal.pone.0179461. 10.1371/journal.pone.0179461 PMC5491017 28662058

[28] Maruoka N, Tamura Y, Tanbo K, Tachibana S, Kamiyama K, Matsumura E (2015). The effect of introducing the partnership nursing system and problems with its establishment (in Japanese). Ishikawa J of Nurs..

[29] De Vet HC, Terwee CB, Mokkink LB, Knol DL (2011). Measurement in medicine: a practical guide.

[30] Odagiri Y, Ohya Y, Inoue S, Hayashi T, Uchiyama A, Takamiya T, et al. Reliablity and validation of the Japanese version of the measure of workplace social capital scale (in Japanese). San Ei Shi. 2010; 52 Suppl: 631.

[31] Shimazu A, Schaufeli W, Kosugi S, Suzuki A, Nashiwa H, Kato A, et al. Work engagement in Japan: validation of the Japanese version of the Utrecht Work Engagement Scale. Applied Psychology. 2008. 10.1111/j.1464-0597.2008.00333.x.

[32] Maertz CP, Boyar SL. Theory-driven development of a comprehensive turnover-attachment motive survey. Hum Resour Manage. 2012. 10.1002/hrm.20464.

[33] R Core Team. R: A language and environment for statistical computing. Vienna: R Foundation for Statistical Computing; 2017. https://www.R-project.org/. Accessed 5 Mar 2020.

[34] Kline P (2013). Handbook of psychological testing.

[35] Foody M, McGuire L, Kuldas S, O’Higgins NJ. Friendship quality and gender differences in association with cyberbullying involvement and psychological well-being. Front Psychol. 2019. 10.3389/fpsyg.2019.01723. 10.3389/fpsyg.2019.01723 PMC6668631 31396139

[36] Trizano-Hermosilla I, Alvarado JM. Best alternatives to Cronbach’s alpha reliability in realistic conditions: congeneric and asymmetrical measurements. Front Psychol. 2016. 10.3389/fpsyg.2016.00769. 10.3389/fpsyg.2016.00769 PMC4880791 27303333

[37] Landis JR, Koch GG. The measurement of observer agreement for categorical data. Biometrics. 1977. 10.2307/2529310. 843571

[38] De Vet HC, Terwee CB, Knol DL, Bouter LM. When to use agreement versus reliability measures. J Clin Epidemiol. 2006. 10.1016/j.jclinepi.2005.10.015. 10.1016/j.jclinepi.2005.10.015 16980142

[39] De Vet HC, Beckerman H, Terwee CB, Terluin B, Bouter LM (2006). Definition of clinical differences. J Rheumatol..

[40] Norman G. Likert scales, levels of measurement and the “laws” of statistics. Adv Health Sci Educ Theory Pract. 2010. 10.1007/s10459-010-9222-y. 10.1007/s10459-010-9222-y 20146096

[41] Ihara M, Kano Y. A new estimator of the uniqueness in factor analysis*.* Psychometrika. 1986; 10.1007/BF02295595.

[42] Gorostiaga A, Aliri J, Ulacia I, Soroa G, Balluerka N, Aritzeta A, et al. Assessment of entrepreneurial orientation in vocational training students: development of a new scale and relationships with self-efficacy and personal initiative. Front Psychol. 2019. 10.3389/fpsyg.2019.01125. 10.3389/fpsyg.2019.01125 PMC6527836 31139129

[43] Japan Nursing Association. 2019 Survey on the actual situation of hospital nursing (in Japanese). 2020. https://www.nurse.or.jp/up_pdf/20200330151534_f.pdf. .

[44] Japan federation of Medical Worker's Unions. 2017 Survey on labor situation of nursing staff (in Japanese). 2018. http://irouren.or.jp/research/75dcde07a35d16c7889d5cc57f2026e143ced7e7.pdf. .

[45] Oksanen T, Kawachi I, Kouvonen A, Takao S, Suzuki E, Virtanen M, et al. Workplace determinants of social capital: cross-sectional and longitudinal evidence from a Finnish cohort study. PLoS One. 2013. 10.1371/journal.pone.0065846. 10.1371/journal.pone.0065846 PMC3679109 23776555

